# Insect oviposition preference between *Epichloë*‐symbiotic and *Epichloë*‐free grasses does not necessarily reflect larval performance

**DOI:** 10.1002/ece3.6450

**Published:** 2020-06-01

**Authors:** Miika Laihonen, Kari Saikkonen, Marjo Helander, Toomas Tammaru

**Affiliations:** ^1^ Biodiversity Unit University of Turku Turku Finland; ^2^ Department of Biology University of Turku Turku Finland; ^3^ Institute of Ecology and Earth Sciences University of Tartu Tartu Estonia

**Keywords:** *Coenonympha hero*, defensive mutualism, *Epichloë*, fungal endophytes, herbivory

## Abstract

Variation in plant communities is likely to modulate the feeding and oviposition behavior of herbivorous insects, and plant‐associated microbes are largely ignored in this context. Here, we take into account that insects feeding on grasses commonly encounter systemic and vertically transmitted (via seeds) fungal *Epichloë* endophytes, which are regarded as defensive grass mutualists. Defensive mutualism is primarily attributable to alkaloids of fungal origin. To study the effects of *Epichloë* on insect behavior and performance, we selected wild tall fescue (*Festuca arundinacea*) and red fescue (*Festuca rubra*) as grass–endophyte models. The plants used either harbored the systemic endophyte (E+) or were endophyte‐free (E−). As a model herbivore, we selected the *Coenonympha hero* butterfly feeding on grasses as larvae. We examined both oviposition and feeding preferences of the herbivore as well as larval performance in relation to the presence of *Epichloë* endophytes in the plants. Our findings did not clearly support the female's oviposition preference to reflect the performance of her offspring. First, the preference responses depended greatly on the grass–endophyte symbiotum. In *F. arundinacea*, *C. hero* females preferred E+ individuals in oviposition‐choice tests, whereas in *F. rubra*, the endophytes may decrease exploitation, as both *C. hero* adults and larvae preferred E− grasses. Second, the endophytes had no effect on larval performance. Overall, *F. arundinacea* was an inferior host for *C. hero* larvae. However, the attraction of *C. hero* females to E+ may not be maladaptive if these plants constitute a favorable oviposition substrate for reasons other than the plants' nutritional quality. For example, rougher surface of E+ plant may physically facilitate the attachment of eggs, or the plants offer greater protection from natural enemies. Our results highlight the importance of considering the preference of herbivorous insects in studies involving the endophyte‐symbiotic grasses as host plants.

## Graphical abstract

In this study, we addressed the question how insect oviposition preference reflects larval performance in a satyrine butterfly when the host plant harbors a fungal endophyte. Against predictions, we found no clear association between oviposition preference and larval performance. On top of that and against defensive mutualism hypothesis, endophytic plants could attract adult insect females and our results suggest that the responsible cue could be of nonchemical nature.

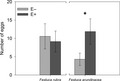

## 1 INTRODUCTION

Microbes are ubiquitous and abundant residents of plants, with a demonstrated ability to affect plants' exploitability by herbivores (Biere & Honders, 2006; Partida‐Martinez & Heil, 2011; Saari, Helander, Faeth, & Saikkonen, 2010; Shikano, Rosa, Tan, & Felton, 2017). Thus, insect–plant interactions cannot be fully understood without taking into account plant‐associated microorganisms (Barbosa, Krischick, & Jones, 1991; Price et al., 1980; Saikkonen, Faeth, Helander, & Sullivan, 1998; Shikano et al., 2017). However, similar to other biotic interactions, microbe–plant interactions are often species‐specific, labile, and context dependent (Ahlholm, Helander, Lehtimäki, Wäli, & Saikkonen, 2002; Clay, 1988; Saikkonen et al., 1998; Saikkonen, Saari, & Helander, 2010; Shikano et al., 2017). This variability should be taken into account in multispecies studies, as it has been shown to affect insects' behavior and performance, which in turn has population‐level consequences (Biere & Honders, 2006; Saikkonen et al., 2010; Shikano et al., 2017; Tack & Dicke, 2013).

In this study, we focus on the fungal endophytes that live asymptomatically within plant foliage (Wilson, 1995) and on how they modulate herbivore behavior and performance. Foliar endophytes are abundant and taxonomically diverse. Virtually all plant species studied to date harbor numerous horizontally transmitted endophytic microfungi, usually causing highly restricted local single spore‐origin infections. They can be dispersed via air, rain splashes, and animal vectors, from senescent and abscised previous season's leaves. These endophytes are ubiquitous including, for example, latent pathogens and dormant saprophytes during their extended asymptomatic periods in their life cycles (Clay, 1988; Saikkonen et al., 1998). In contrast to other taxa, *Epichloë* species (Hypocreales: Clavicipitaceae) form systemic and lifelong endophytic symbiosis with cool‐season grasses. Due to systemic growth throughout the aboveground parts of the host, including developing inflorescences, the transmission of the *Epichloë* occurs mainly vertically from mother plants to offspring via seeds. Similar to other endophytes, *Epichloë* species are thought to have a pathogen or saprotroph ancestry and to have evolved from such by means of an extended latent phase in their life cycle (Rodriguez, White, Arnold, & Redman, 2009; Saikkonen, Helander, & Faeth, 2004; Saikkonen, Wäli, Helander, & Faeth, 2004). In the case of the *Epichloë* species, a reduction of virulence has led to limited or complete loss of opportunities for contagious spread by spores (Rodriguez et al., 2009; Saikkonen, Helander, et al., 2004; Saikkonen, Wäli, et al., 2004).

The tightly linked fitness of the *Epichloë* species and their host grasses has commonly been assumed to drive evolution toward mutually beneficial cooperation, primarily by an antiherbivore defense provided by the fungus (Clay, 1990; Clay & Schardl, 2002; Saikkonen et al., 1998; Saikkonen, Helander, et al., 2004). Empirical evidence supports the idea of defensive mutualism (Clay, 1987, 1988, 2009; Saikkonen et al., 2010). The host plant provides the fungus with nutrition, shelter, and distribution via seeds, whereas the *Epichloë* species have been demonstrated to be able to deter a wide range of herbivores, from insects to vertebrates. Defensive mutualism is commonly recognized to be based on alkaloids of fungal origin that are added to the chemical repertoire of the host grasses (Deshmukh, Verekar, & Bhave, 2015; Saikkonen, Gundel, & Helander, 2013; Saikkonen et al., 2010; Saikkonen, Young, Helander, & Schardl, 2016; Schardl, 1996), particularly in high‐nutrient agro‐environments (Ahlholm et al., 2002; Faeth, Helander, & Saikkonen, 2004; Helander et al., 2016; Rodriguez et al., 2008; Saikkonen, 2000; Saikkonen et al., 2010; Saikkonen, Wäli, et al., 2004). The *Epichloë* derived bioactive alkaloids were discovered in the late 1970s. Since then, the majority of research on defensive mutualism has focused on these alkaloids, largely ignoring the other chemotypic diversity of endophyte–grass interactions and the other possible associated transformations in the host grasses' traits. Such endophyte‐derived changes in plants may include higher silica content in *Epichloë*‐symbiotic plants, which can manifest as physical changes in a plant that may translate to better herbivore resistance (Coughenour, 1985; Huitu et al., 2014).

In the present study, we examined how *Epichloë* endophytes affect host preference and performance in the different developmental stages of an insect herbivore. Until recently, studies on the interactions among endophytes, plants, and insects have paid little attention to whether the insect preference is adaptive (Shymanovich & Faeth, 2018; Shymanovich, Musso, Cech, & Faeth, 2019). In addition to survival, feeding preference, and performance studied elsewhere (Shymanovich & Faeth, 2018; Shymanovich et al., 2019), we examined association between the preference of a female insect and the performance of her offspring. By using endophyte‐symbiotic (E+) and endophyte‐free (E−) conspecific *Festuca rubra* L. and *F. arundinacea* Schreb. plants, we experimentally examined how the presence of *Epichloë* in the host plant affects the females' oviposition preference, the larval feeding preference, and the larval performance of the scarce heath butterfly (*Coenonympha hero* L.). We assumed that females lay their eggs on the host plant on which their offspring perform the best (Gripenberg, Mayhew, Parnell, & Roslin, 2010). Because the *Epichloë* species associated with our model plant species are known to be able to produce herbivore‐deterring and insecticidal alkaloids (Helander et al., 2016; Saikkonen et al., 2013; Vázquez de Aldana, Leinonen, Zabalgogeazcoa, Helander, & Saikkonen, 2020), we hypothesized that if the grasses and *C. hero* have a long co‐evolutionary history, and the associated *Epichloë* species negatively affect the fitness of *C. hero*, the butterfly should have evolved to be able to detect the fungus in the host grass and avoid E+ plants. Alternatively, the preference and performance should not necessarily match if the butterfly is not highly dependent on endophyte‐symbiotic grasses as a food source. *Coenonympha hero* larvae are known to feed on grasses but the relative importance of different grass species as larval host plant is poorly known. Thus, the results enlighten the co‐adaptive history of the species.

## 2 MATERIALS AND METHODS

We used two perennial fescue grass species, tall fescue (*F. arundinacea* Schreb.) and red fescue (*F. rubra* L.), both native to Europe, occurring naturally and as forage grasses widely throughout the northern temperate region. *Festuca arundinacea* is a robust bunchgrass harboring the asexual fungal endophyte *Epichloë coenophiala* (Leuchtmann, Bacon, Schardl, White, & Tadych, 2014). *Festuca rubra* is a more loosely growing, fine‐leaved fescue that often harbors the symbiotic endophyte *E. festucae* (Leuchtmann et al., 2014).

The seeds for this experiment were collected from plants in two established experimental settings (Helander et al., 2016; Leinonen, Helander, Vazquez‐de‐Aldana, Zabalgogeazcoa, & Saikkonen, 2019) at Ruissalo Botanical Garden (WGS 60.43 N, 22.17 E). The *F. arundinacea* plants were originally collected from the Åland Islands in southwest Finland. Roughly 95% of the *F. arundinacea* plants in the Åland Islands have symbiotic *Epichloë* endophytes and produce lysergic acids, lolines, and high levels of ergot alkaloids (Helander et al., 2016; Saari et al., 2010). The endophyte‐free (E−) *F. arundinacea* plants from which the seeds were collected for this experiment had been originally produced manipulatively by heat‐treating the seeds of the endophyte‐symbiotic (E+) plants (Wäli, Helander, & Saikkonen, 2014). *Festuca rubra* mother plants were originally collected from the wild in the Salamanca area, in inland Spain. The E+ plants are known to produce high concentrations of insect‐deterring peramine and insecticidal ergovaline (Vázquez de Aldana et al., 2020).

For the present study, we collected seeds from four E+ and four E− free‐pollinated mother plants of red fescue and tall fescue. As fescues are both wind‐ and cross‐pollinated, we assumed that the offspring of an individual mother plant were half‐siblings. Thus, this study was based on four half‐sibling groups in both E+ and E− plants of both species. The minimum of three pots (8 cm × 8 cm in size; each with more than five individuals) of each half‐sibling group (50 pots in total, later referred to as “plants”) were grown in greenhouse conditions (stable ~15°C; ambient light; potting soil: Biolan for saplings; fertilizer: 1 dl of Biopon Rose every other week according to instructions) at Ruissalo Botanical Garden for 2 months before they were transferred to growth chambers [16 hr: ~22°C, light; 8 hr: ~16°C, dark] at the University of Tartu, Estonia. All plants were cut to a height of 5 cm 2 weeks before the first experiments to induce tillering so as to obtain more plant material.

We double‐checked the endophyte presence in the study plants both from the collected seed material and from the leaves of the growing study plants. This was necessary due to the possibility that the endophytes might fail to transmit to the next host generation or die before the seeds germinated. The seeds were softened by soaking them for 12 hr in a solution of sodium hydroxide and ethanol [95 ml water, 5 ml ethanol, and 2.5 g NaOH], after which they were microscopically examined. The hyphae of the endophytic fungus can be detected between the embryo cells in the seeds of the endophyte‐symbiotic plants. To identify whether the growing plants had the systemic fungus, surface‐sterilized leaves [70% ethanol, 30 s; 4% NaOCl, 3 min; 70% ethanol, 15 s] were cut into pieces and placed on Petri dishes containing potato dextrose agar (2%). In the E+ plants, the *Epichloë* hyphae grew out of the cut edges of the leaves.

As the model herbivore, we chose the scarce heath (*C. hero* L.). This small satyrine butterfly is distributed from Central and Northern Europe to northwestern Asia. In Northern Europe, its single annual generation flies from late May to early July. The imago feeds on the nectar of various meadow plants, not including wind‐pollinated grasses. The female lays eggs one by one, attaching them to vegetation. Normally a female can lay more than 100 eggs during its lifetime and more than twenty in one day. The newly hatched larvae are able to move short distances, which enables them to explore their surroundings for suitable host plants (Bonelli, Canterino, & Balletto, 2010; Lindman, Johansson, Gotthard, & Tammaru, 2013; Tiitsaar, Kaasik, Lindman, Stanevits, & Tammaru, 2016). In nature, the larvae feed on many grasses and sedges including *Carex*, *Calamagrostis,* and *Festuca* species (Tiitsaar et al., 2016; Tolman & Lewington, 1997). It has been detected on *F. rubra*, but it was as yet unknown whether they were able to use the leaves of the more robust *F. arundinacea*.

The adult female insects used in the experiments were caught from the wild in Estonia in the beginning of June 2016:46 individuals from 4 locations near Tartu, 3 individuals from Märjamaa, and 2 from the Pärnu area. In its natural habitats, *C. hero* may encounter both of the grass species used in this study, as they are abundant in Estonia. When they were not used in the experiments, the wild‐captured females were kept individually in small jars in a refrigerator. Food (sugar solution) for them was always present on tissue in the jars. The larvae in the feeding experiments were first‐generation, laboratory‐born offspring of the wild females used in the oviposition experiments. The eggs were kept in small jars and the newly hatched larvae were not fed before the experiments so as to avoid biasing their preference for the food given beforehand.

### 2.1 Oviposition‐choice test

To examine the oviposition preference of the *C. hero* females relative to the presence of endophytes, we conducted trials that introduced the butterflies individually to terrarium boxes (35 cm × 25 cm × 20 cm) that gave them the choice between E+ and E− *F. arundinacea* or *F. rubra* plants (*N_arundinacea_* = 23 trials; *N_rubra_* = 24 trials). The pots with plants were placed under the terraria, and the aboveground parts of the potted E+ and E− plants were inserted in opposite corners of the terrarium through holes made in the bottom of the box. Both plants inside the same terrarium were approximately equal in biomass and similar in appearance. We investigated all possible combinations of E+ and E− half‐sibling plant groups. During the experiment, some of the plant individuals were used twice with different counterparts making all the plant combinations unique. The positions of the E− and the E+ plants were alternated in the terraria, and an adjustable table lamp was put above each to provide the females with light and warmth (about 27°C inside the terraria). The light was on daily from approximately 8 a.m. to 8 p.m. One female at a time was kept in a terrarium with the E− and E+ plants until it had laid several (>5) eggs on the plants. A different female was used in each trial. After the end of the experiment, we recorded the number of eggs attached to each plant.

### 2.2 Oviposition‐rate test

To determine the attractiveness of the E− and E+ plants as oviposition substrates, we conducted trials to measure the oviposition rate of the *C. hero* females exposed to a single plant, either E− or E+, for a fixed amount of time (single‐substrate design). We assumed that the oviposition rate indirectly reflects the host's quality, because the female should save her eggs if there is no preferred plant available (Tammaru, Kaitaniemi, & Ruohomäki, 1995). In contrast, the oviposition rate should be high if an ideal plant is present. The number of eggs laid in a fixed period provided an approximation of the plant's suitability from the perspective of the ovipositing female (Javoiš & Tammaru, 2004, 2006).

The experiment was conducted in growth chambers [17 hr: ~27°C, light; 7 hr: ~20°C, dark]. The adult females were placed in transparent plastic boxes (1.5 L) into which the shoots of potted E+ or E− plants of *F. arundinacea* or *F. rubra* (*F. arundinacea*: *N*_E−_ = 30; *N*_E+_ = 33 and *F. rubra*: *N*_E−_ = 35; *N*_E+_ = 34) had been inserted through a hole in the bottom. Small holes in the lids let excess humidity evaporate from the box. After 21 hr, the females were removed from the experimental arena, and the eggs they had laid were counted. Each female was used in this experiment up to four times if her condition allowed it. Each was exposed to all four plant types (two species with both E+ and E− individuals), and the first and second plants were of the same species before the plant species was switched. The starting plant type was randomly chosen for each female to take into account the possible effect of previous experience on oviposition decisions.

### 2.3 Food‐plant preference test

To examine larval food preference, we conducted trials that enabled the mobile, newly hatched *C. hero* larvae to choose between E− and E+ plants. The leaves of living, potted E+ and E− plants were inserted from opposite directions between the lid of a Petri dish, and a single larva was placed in the center of the Petri dish (*N_arundinacea_* = 74; *N_rubra_* = 78 trials). After 18 hr, the experiment was terminated, and the leaves were examined for signs of larval consumption (missing leaf parts) (see Lindman et al., 2013 for a similar design).

### 2.4 Larval growth and survival test

To compare the quality of the E− and E+ plants for larvae, we conducted a no‐choice rearing experiment that mimicked the situation in which the larvae have no possibility of switching to another plant after hatching. We measured the growth and survival of the larvae that were feeding on either the E− or E+ plants of *F. rubra* or *F. arundinacea* (*F. rubra*: *N*_E−_ = 120, *N*_E+_ = 125; *F. arundinacea*: *N*_E−_ = 115, *N*_E+_ = 110) in a growth chamber. The leaves of a potted, living plant were placed so as to grow through the mesh tubes (12 cm × 10 cm) in which the larvae were reared. The amount of plant material enclosed in the tubes was more than sufficient to support all the larvae. These mesh tubes were closable at each end and had a wooden ring in the middle that kept the tube from collapsing. Each mesh tube contained five newly hatched and active larvae from the same population. After 2 weeks, the living larvae were weighed using a precision balance, and the weight was used as an index of individual performance. Any larvae missing from the mesh tubes were counted as having died, and survival rate was calculated accordingly.

### 2.5 Statistical analyses

For both oviposition tests (choice and rate, see above), we used a general linear mixed effect model (later “glm”) to test if the presence of the endophytes (categorical explanatory variable: E+ and E− plants) explained the number of eggs laid (response variable) on particular plants. As the same *C. hero* butterfly was tested up to four times, the ID number of a female (nested within insect population) was used as a random factor to account for repeated observations. We compared the plant types (two species with both E+ and E− individuals) using Tukey tests. In the oviposition‐choice test, we considered only the eggs that were attached to the plants (~90% of all eggs), and *F. arundinacea* and *F. rubra* were analyzed separately. Each female (= trial) was treated as an independent observation. In the oviposition‐rate test, the response variable (number of eggs) was square‐root‐transformed before the analysis to achieve the normal distribution.

In the larval food‐plant preference test, we tested *F. arundinacea* and *F. rubra* separately as to whether either E+ or E− plants (explanatory variable) were consumed differently. Most larvae had consumed only one leaf type (either E− or E+), so it was reasonable to treat the response variable as a binary one (the larvae consumed either more E− (0) or E+ (1)); an ordinary chi‐square test was performed to compare the preference pattern to the default 1:1 ratio. Each larva (= trial) was treated as an independent observation. In the growth and survival experiment, we applied mixed glm to test whether the presence of endophytes explained larval survival and/or biomass. Mesh tube's ID was used as a random factor to account for the effect of a common environment shared by the larvae within a tube. We used Tukey tests to compare larval biomass between the plant types. For larval survival, we used a generalized linear model for binary data. To statistically test the difference in mortality between the two plant species, a two‐sample *t* test was conducted using the number of surviving larvae in a mesh tube. The analyses were conducted using Statistical Analysis Software (SAS).

## 3 RESULTS

### 3.1 Oviposition‐choice test

**FIGURE 1:**
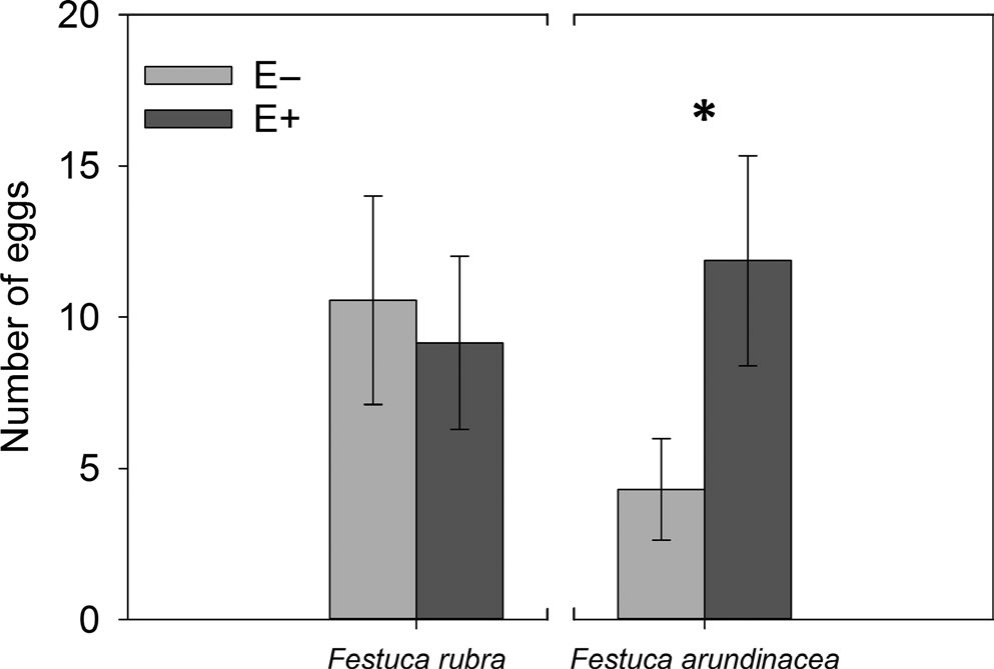
Numbers of eggs laid by *Coenonympha hero* on *Festuca arundinacea* plants in terraria in which two plants were offered simultaneously. Endophyte‐symbiotic (E+) plants were clearly preferred over endophyte‐free (E−) ones whereas there was no preference in *Festuca rubra*.*Festuca rubra*: *N*_plant–plant–insect combinations_ = 24. ẋ_E−_ = 10.56, ẋ_E+_ = 9.15; *Festuca arundinacea*: *N*_plant–insect combinations_ = 23. ẋ_E−_ = 4.3, ẋ_E+_ = 11.9. Note that direct comparisons between plant species are not viable. Error bars indicate 95% confidence intervals. The asterisk indicates statistically significant difference

The *C. hero* females revealed their preference for the E+ *F. arundinacea* plants by ovipositing more than 70% of their eggs onto them (mixed glm: *F*_1;44_ = 16.50; *p* = .0002) (Figure 1). In contrast, in the case of *F. rubra* plants, the females showed no preference for either the E+ or the E− plants (mixed glm *F*_1;43_ = 0.48; *p* = .49) (Figure 1).

### 3.2 Oviposition‐rate test

**FIGURE 2:**
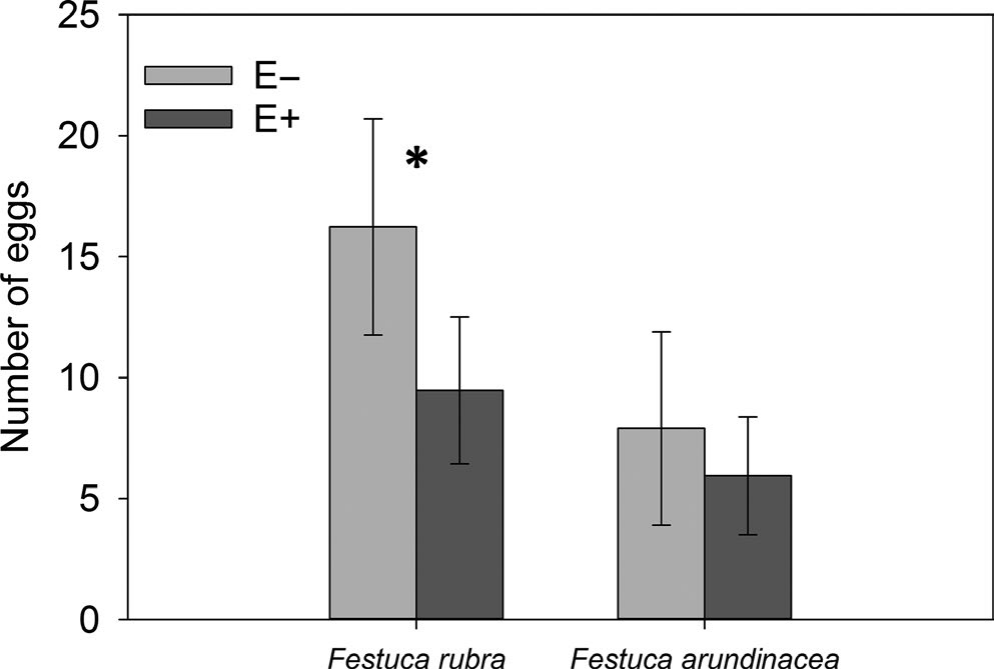
Numbers of eggs laid by *Coenonympha hero* in the presence of endophyte‐symbiotic (E+) and endophyte‐free (E−) *Festuca arundinacea* and *Festuca rubra* plants in the oviposition‐rate experiment. Females laid eggs at highest rate (approximation of preference) when an E− *Festuca rubra* plant was presented. *N_F. rubra_*_ E−_ = 35, *N_F. rubra_*_ E+_ = 34, *N_F. arundinacea_*_ E−_ = 30, *N_F. arundinacea_*_ E+_ = 33. ẋ*_F. rubra_*_ E−_ = 16.2, ẋ*_F. rubra_*_ E+_ = 9.5, ẋ*_F. arundinacea_*_ E−_ = 7.9, ẋ*_F. arundinacea_*_ E+_ = 6.0. Error bars indicate 95% confidence intervals. The asterisk indicates statistically significant difference

On the *F. arundinacea* plants, the females laid their eggs at the same rate on both the E+ and the E− plants (mixed glm: *F*_1;46.4_ = 0.50; *p* = .48). In contrast, on the *F. rubra* plants, the females laid more than 75% more eggs on the E− plants (mixed glm: *F*_1;58.9_ = 6.87; *p* = .011) (Figure 2). The E− *F. rubra* plants were also preferred over the *F. arundinacea* plants (pairwise comparisons: with E− *F. arundinacea*: *t*_63_ = 2.30, *p* = .010, with E+ *F. arundinacea*: *t*_66_ = 3.94, *p* = .0008) (Figure 2). There was no difference in preference between E+ *F. rubra* and *F. arundinacea* plants.

### 3.3 Food‐plant preference test

**FIGURE 3:**
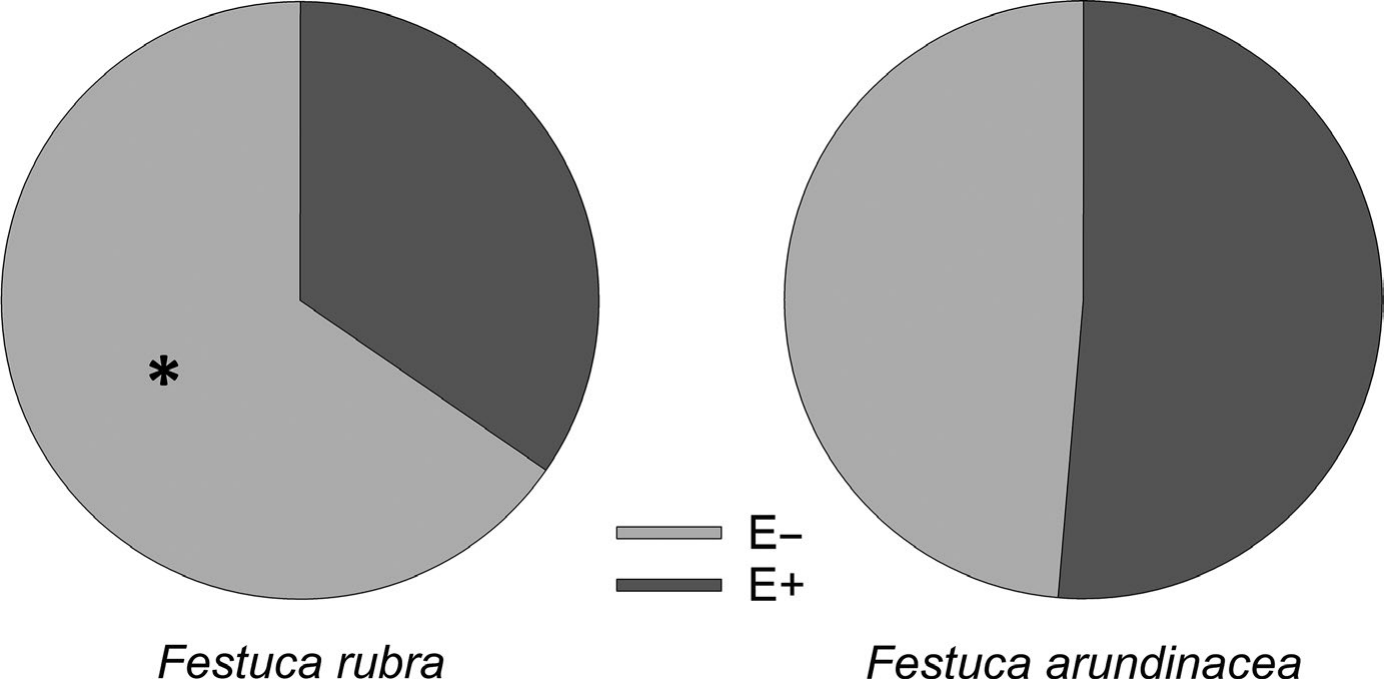
Distribution of *Coenonympha hero* larvae on *Festuca arundinacea* and *Festuca rubra* in food‐plant preference test. On the *F. rubra*, the endophyte‐symbiotic plants (E+) were preferred over the endophyte‐free plants (E−). *N_F. arundinacea_* = 74*, N_F. rubra_* = 78. The asterisk indicates statistically significant difference

The larvae did not show a preference for either the E− or E+ plants of the *F. arundinacea* ( $\chi_{1}^{2}$ **=** 0.054; *p* = .81). In contrast, on the *F. rubra* plants, the larvae chose the E− plants roughly twice as often ( $\chi_{1}^{2}$ **= **7.38; *p* = .0066) (Figure 3).

### 3.4 Larval growth and survival test

Larval survival was nearly five times higher on *F. rubra* (44% survived) than on *F. arundinacea* (9% survived) plants (two‐sample *t* test: *t*_72.805_ = −6.75, *p* = .0001). However, we found no effects of endophyte symbiosis on larval survival or growth on either grass species. On the *F. arundinacea* plants*,* the mortality of the *C. hero* larvae on both E− and E+ plants was equally high (E−: %_survival_ = 8.7, E+: %_survival_ = 9.1; generalized linear model for binary data: *F*_1;59.34_ = 0.02; *p* = .90). Similarly, the endophyte did not affect the survival of the *C. hero* in the *F. rubra* plants (E−: %_survival_ = 46.7, E+: %_survival_ = 40.8; generalized linear model for binary data: *F*_1;40.94_ = 0.47; *p* = .49). The growth of *C. hero* did not differ significantly among any plant types (weights after 2 weeks: ẋ*_F. rubra_*_ E−_ = 2.97 mg, ẋ*_F. rubra_*_ E+_ = 2.54 mg, ẋ*_F. arundinacea_*_ E−_ = 1.98 mg, ẋ*_F. arundinacea_*_ E+_ = 2.54 mg; *F*_3;123_ = 2.25; *p* = .086).

## 4 DISCUSSION

We found that endophytic *Epichloë* in host plants affects the choices of both ovipositing females and the larvae of a butterfly. However, the effects depended on the interacting endophyte and/or plant species comprising the symbiotum. We conclude that E− *F. rubra* plants provide a stronger oviposition stimulus than E+ counterparts, but when both plants are simultaneously presented, the females of *C. hero* do not distinguish between the substrates at the moment of oviposition. However, in *F. arundinacea*, females are similarly ready to oviposit on either E+ or E− plant but they prefer the E+ as the oviposition substrate if they can choose between those two plants. Larval performance was not affected by the presence of the fungus. Instead, host plant species was the primary determinant of larval performance: *C. hero* is clearly better adapted to the fine‐leaved *F. rubra* than to the more robust *F. arundinacea*. Furthermore, although we did not explicitly compare the two host plant species in multiple choice trials, the host plant species preference and performance appeared to match. The rates of oviposition and larval survival for *C. hero* are 1.9 and 4.9 times higher on the *F. rubra* than on the *F. arundinacea* plants, respectively, indicating that the females oviposit more readily on the plant species better supporting the growth of their offspring.

To some extent, these findings contrast with the defensive mutualism hypothesis, which states that *Epichloë* endophytes negatively affect herbivores via deterring and/or insecticidal alkaloids (Clay, 1987, 1988, 2009; Saikkonen et al., 2010). For example, the E+ *F. rubra* plants used contain substantial amounts of peramine and ergot alkaloids (Vázquez de Aldana et al., 2020), but the larvae survived and grew equally well on the E− and the E+ plants. Still, the defensive mutualism hypothesis remains viable, as it seems to work on the level of insect preference in the case of *F. rubra*. Both *C. hero* females and larvae preferred the E− plants. One explanation for the lack of negative effects on larval performance may be the short exposure times in our bioassays. During the 2 weeks of the experiment, the weight accumulation of the larvae was roughly 15% less on the E+ *F. rubra* plants than on the E− conspecifics but the difference did not attain statistical significance. Longer experiments capturing the entire life cycle might have yielded a better detectable difference in performance. Alternatively, the detected avoidance of E+ *F. rubra* plants does not necessarily indicate a specific adaptation. The larvae of *C. hero* are polyphagous herbivores of grasses (Tiitsaar et al., 2016), which decreases the probability of species‐specific adaptations to particular host plant species and/or associated symbiotic endophytes. If so, the detected avoidance of E+ *F. rubra* plants may mirror a general and evolutionarily old adaptation of *C. hero* to avoid insect‐deterring alkaloids rather than an outcome of a specific species interaction.

The findings with *F. arundinacea* support the lack of species‐specific adaptations in the studied system. In contrast to the hypothesis of defensive endophyte–grass mutualism (Clay, 1988, 2009; Saikkonen et al., 2010), the females preferred to oviposit on E+ *F. arundinacea* plants although our previous studies have detected substantial amounts of ergot alkaloids, lysergic acid, and lolines in the same plant origins (Helander et al., 2016). Lolines in particular have anti‐invertebrate properties (Clay & Schardl, 2002; Saikkonen et al., 2016). Thus, the result that the females prefer to oviposit on the E+ plants, and that overall fewer than 10% of larvae survived on the *F. arundinacea* plants regardless of endophyte infection, suggest that the females' behavior was driven by grass traits that are associated with the endophytes but are irrelevant for next‐generation larvae. The trait may include, for example, altered volatile emissions or foliar silicon content, which are commonly considered to modulate trophic interactions (Coughenour, 1985; Dyer et al., 1991; Huitu et al., 2014; Karban & Baldwin, 2007; Li, Blande, Gundel, Helander, & Saikkonen, 2014).

Interestingly, our results suggest that the endophyte modulated *F. arundinacea* traits may work as a positive oviposition cue for an insect herbivore. This can be adaptive for the insect if the E+ plants constitute a favorable oviposition substrate for reasons other than the nutritional quality of the plants, and the larvae are not bound to feed on the plant chosen by their mother. Indeed, *C. hero* larvae are capable of moving from host to host (Tiitsaar et al., 2016, this study), and thus, we may assume that females need not choose host plants based on plant chemistry but instead rely on physical leaf attributes that increase the chances of egg survival (Eilers, Petterson, & Öckinger, 2013; Krämer, Kämpf, Enderle, Poniatowski, & Fartmann, 2012; Lawson, Bennie, Hodgson, Thomas, & Wilsonet, 2014; Tammaru et al., 1995; Tiitsaar et al., 2016). In the case of *F. arundinacea*, *Epichloë* symbiosis can promote ovipositing and survival of eggs in two ways. First, endophytes increase silica accumulation in plants, which affects physical parameters of plant surface (Huitu et al., 2014) in such a way that insect eggs can be better attached to it. Second, endophytes alter host plant growth, thus possibly enhancing the microclimatic conditions for the developing eggs (Krämer et al., 2012; Lawson et al., 2014; Rodriguez et al., 2008; Saikkonen, Helander, et al., 2004; Saikkonen, Wäli, et al., 2004; Schardl, 1996). Another possible scenario is that *C. hero* female chooses the oviposition substrate to protect its progeny from predators that could be more strongly affected by the *Epichloë* derived alkaloids (Jani, Faeth, & Gardner, 2010; Omacini, Chaneton, Ghersa, & Müller, 2001; Saari, Richter, Robbins, & Faeth, 2014).

These results reveal the following broader outlooks. First, the results emphasize the complexity of the grass–herbivore interactions mediated by *Epichloë* endophytes. The endophytes may differently affect the different life stages of the herbivorous insects associated with different plant species. Second, even a negative association between insect oviposition preferences and offspring performance could persist in nature. Third, the identified oviposition preference toward E+ *F. arundinacea* plants may affect the distribution of *C. hero* and possibly some other insect herbivores and lead to increased herbivory pressure on the E+ plants in plant communities (Tack & Dicke, 2013). Therefore, future studies on hypotheses regarding defensive mutualism between endophytes and their host grasses should explicitly consider the behavioral traits of the insect herbivores feeding on the endophyte‐symbiotic plants as well as explore the effects of these endophytes to higher trophic level interactions.

## CONFLICT OF INTEREST

None declared.

## AUTHOR CONTRIBUTIONS

**Miika Laihonen:** Conceptualization (equal); Data curation (lead); Formal analysis (lead); Investigation (lead); Methodology (equal); Visualization (equal); Writing‐original draft (lead); Writing‐review & editing (equal). **Kari Saikkonen:** Conceptualization (equal); Funding acquisition (equal); Methodology (equal); Project administration (equal); Resources (equal); Supervision (equal); Writing‐original draft (supporting); Writing‐review & editing (equal). **Marjo Helander:** Conceptualization (equal); Funding acquisition (equal); Methodology (equal); Project administration (equal); Resources (equal); Supervision (equal); Writing‐original draft (supporting); Writing‐review & editing (equal). **Toomas Tammaru:** Conceptualization (equal); Data curation (supporting); Formal analysis (supporting); Funding acquisition (equal); Investigation (supporting); Methodology (equal); Project administration (equal); Resources (equal); Supervision (equal); Writing‐original draft (supporting); Writing‐review & editing (equal).

## Data Availability

All appropriate data have been archived to Dryad: https://doi.org/10.5061/dryad.5qfttdz2c
